# Effects of Different Storage Media, Temperature, and Time on Osteoblast Preservation in Autogenous Bone Grafts: A Histomorphometrical Analysis

**DOI:** 10.30476/DENTJODS.2020.81808.0

**Published:** 2020-09

**Authors:** Hamidreza Arabiun, Hossein Bordbar, Seifollah Dehghani Nazhvani, Reyhaneh Ebrahimi, Ehsan Aliabadi, Ilnaz Ghanbari

**Affiliations:** 1 Dept. of Oral and Maxillofacial Surgery, School of Dentistry, Shiraz University of Medical Sciences, Shiraz, Iran; 2 Dept. of Anatomical Sciences, Histomorphometry, Stereology Research Center, Shiraz University of Medical Sciences, Shiraz, Iran; 3 Dept. of Clinical Science, School of Veterinary Medicine, Shiraz University, Shiraz, Iran; 4 Dept. of Periodontology, School of Dentistry, Shiraz University of Medical Sciences, Shiraz, Iran; 5 Postgraduate Student, Dept. of Oral and Maxillofacial Surgery, School of Dentistry, Shiraz University of Medical Sciences, Shiraz, Iran

**Keywords:** Autogenous bone graft, Osteoblast, Extracorporeal preservation, Osteogenic cells, Histomorphometrical analysis

## Abstract

**Statement of the Problem:**

Autogenous bone graft is the gold standard for bone reconstruction. Osteogenic cells must be kept viable in graft for a successful procedure. In extracorporeal preservation of grafts during surgery, three different factors may influence the quality of grafts. These factors include temperature, storage medium, and time interval.

**Purpose:**

In this study, we evaluated the effects of different storage media, temperatures, and times on osteoblast count in autogenous bone grafts, preserved extracorporeally.

**Materials and Method:**

Samples were obtained from iliac crest region in a goat. The grafts were preserved in 36 groups of different storage time, temperature, and medium. Samples were histomorphometrically analyzed to determine osteoblast count as the criteria of graft quality.

**Results:**

In almost all samples, room temperature was the most and incubator was the least favorable storage temperatures. In grafts preserved in room temperature, no difference was noted between normal saline and Ringer’s lactate solution and in almost all of the samples autologous blood and dry environment were more favorable media than Ringer’s lactate solution. The effect of storage time was highly depended on the combination of temperature and solution.

**Conclusion:**

The results demonstrated that for preserving as many osteoblasts as possible in bone grafts, the best temperature was room temperature and the least favorable temperature was incubator. In addition, when bone fragments were preserved in room temperature, the best medium for graft storage was blood, which showed better results than normal saline and Ringer’s lactate solution.

## Introduction

Autogenous bone graft is considered the best method and gold standard for bone defect reconstruction, as it provides three basic factors including osteoinductive molecules, a proper scaffold, and osteogenic cells. It is crucial to keep graft osteogenic cells viable because they are responsible for new bone formation after transplantation [ 1 ]. Although it is highly recommended to implant the autogenous grafts immediately after harvest, it may not be possible. As a result, the bone graft must be stored for a certain amount of time extracorporeally [ 2 , 3 ]. 

Several storage media have been introduced for graft preservation including normal saline solution, Ringer’s lactate solution, distilled water, different culture media [ 4 ], and autologous blood derived products including whole blood, plasma, serum, platelet-rich-plasma (PRP) [ 5 ], platelet-poor-plasma (PPP) [ 6 ], and so on.

Normal saline solution and Ringer’s lactate solution are the most common infusion fluids in operation room settings. Normal saline solution is also the most common solution used in surgeries as coolant and irrigation [ 4 ]. Most surgeons also consider whole blood a proper medium for bone graft maintenance and it can easily be obtained during surgery [ 5 ]. Although air exposure is considered the most inappropriate medium for bone graft maintenance, in clinical practice the grafts are often stored in dry environments [ 7 ].

Rocha et al. [ 8 ] studied the effects of preserving bone grafts for 30 minutes in different storage media including normal saline solution, PPP, and dry environment in comparison with a control group, which was implanted immediately. The results showed more empty lacunae in the dry group when compared with the control group, the saline group, or the PPP group. No significant difference was reported in the count of empty lacunae between the saline group, the PPP group, and the control group.

Another factor is preservation temperature. There are three temperature ranges usually possible in operation rooms, during surgeries. These include cold preservation (2-8^°c^), room temperature (18-24^°c^), and incubator (37^°c^). Antonenas et al. [ 9 ] preserved blood stem cells in room temperature and refrigerator for 24 hours, 48 hours, and 72 hours. His study showed a great loss of viable cells in the grafts stored in room temperature (21.9%) compared to those stored in refrigerator (9.4%). Finally, the time interlude that a graft is preserved extracorporeally, can affect the quality of the bone graft. Williams et al. [ 10 ] stored canine femoral condyles at 4^°c^ for 14, 21, and 28 days. His study showed >95% cell viability at 14 days, 75-98% at 21 days, and 65-90% at 28 days of preservation. In this study, we have assessed the effects of three major factors on regenerative potential of autogenous cancellous bone grafts.

## Material and Method

A one-year-old healthy brown female goat (Capraaegagrus hircus) weighing 28 kg was chosen as the animal model. This study was done according to International animal rights with adherence to the animal experiment rules assigned by Shiraz University of Medical Sciences. Before the surgery, 30^cc^ of blood was obtained from right jugular vein under aseptic condition. The blood was mixed with 6^cc^ of sodium citrate 3.8%, as anticoagulant. The blood was transferred to previously sterilized and labeled laboratory tubes assigned for blood as storage medium. Other tubes contained sterile normal saline solution; Ringer’s lactate solution, or they were empty. 

The animal was anesthetized by Ketamine (25mg/ kg) and midazolam (0.2mg/kg) intramuscularly. For local anesthesia, 3.8 ml of lidocaine with epinephrine was injected in surgical site. A single dose of Pen &amp; Strep (2.5mg/kg) was injected intravenously in right jugular vein. The animal was placed on surgery table in sitting position. Surgery was conducted under aseptic condition. The hair on right iliac crest area was shaved and antisepsis was done with aqueous solution of povidone. Animal was covered with sterile drapes and the surgical site was exposed through a perforated drape.

A full-thickness 5cm long incision was placed over right anterior iliac crest with a # 15 blade. Using periosteal elevator, medial and lateral subperiosteal dissection was performed to expose the lateral surface of the iliac crest completely. A 3cm×3cm cortical window was removed by chisel and osteotome to gain access to the cancellous bone. Cancellous blocks were removed by curette and chisel. Blocks were cut by a fine scissor to 180 pieces; the mean volume was 4mm×4mm×4mm. each bone graft sample was transferred to a tube containing storage media.

Copious irrigation of the surgical site with normal saline was done. No active bleeding was noticed. Periosteum, muscle, and fascia were separately sutured with vicryl 4-0. The skin was sutured with Nylon 3-0 and dressing was applied. For studying the effects of three major factors on regenerative potential of autogenous cancellous bone grafts, we used histomorphometrical analysis to evaluate osteoblast count in graft volume unit. These factors included:

1. Different storage media including normal saline solution, Ringer's lactate solution, autologous blood, and dry environment

2. Different storage temperature including cold preservation (2-8^°c^), room temperature (18-24^°c^), and incubator (37^°c^)

3. The time interval that the bone grafts were preserved: 2 hours, 4 hours and 12 hours

The tubes containing specimen and storage medium were distributed evenly in three categories. Each category included 60 tubes and represented storage temperature, including cold preservation, room temperature, and the incubator. Among the 60 tubes in each category, 15 contained normal saline solution, 15 contained Ringer’s lactate solution, 15 contained autologous blood with anticoagulant and 15 tubes did not contain any medium representing the dry environment. Two hours after the harvest time, from each temperature category and storage medium groups, five tubes were selected randomly and the specimens were immediately immersed in 10% formaldehyde solution for 48 hours. Same procedure was done after 4 hours and 12 hours of bone graft preservation. 

Bone graft decalcification was performed by EDTA 4.13%, dehydration by ethanol 95% and embedded in paraffin. We used oriented method in order to obtain isotropic uniform random (IUR) sections. Paraffinized bone grafts were semi-serially sectioned in 20µm thickness (H&amp;E staining) using a microtome.

A video-microscopy system consisting of a microscope (Nikon, E-200, Japan) linked to a video camera (SONY, SSC Dc 18P, Japan), a P4 PC computer and a LG monitor (795 FT plus) was utilized for analysis.

The number of osteoblasts was counted with an optical dissector design for 20µm thick sections. Through this method, the count of osteoblasts in a volume unit of the bone specimen was determined. An unbiased counting frame was superimposed on the image of bone graft section on monitor on average 70-100 microscopic fields, which were selected for every bone graft sample. The counting frame had two borders of inclusion and two borders of exclusion. The initial field was selected randomly out of the sample section; the remaining fields were selected by moving the microscope stage using microscope stage along X- and Y-axis in equal intervals. An oil immersion lens with ×100 magnification was used. To assess the numerical density of osteoblasts, the focus area was also moved on Z-axis. Through traveling on Z-axis and using magnification of 60×, a microcater (Hidenhain MT-12, Germany) which calculated the Z-axis movements was employed. Each plane thickness is 5µm, but the first nuclei that came into focus, were excluded. Within the next 5µm of traveling on Z-axis (height) any nucleolus which came into maximal focus was counted, if located within the counting frame or in contact with the inclusion border and did not touch the exclusion border or the frame. Numerical density of osteoblasts was calculated by the following formula [ 11 ]:

$N_{v}=\frac{\mathrm{ƩQ}}{\mathrm{(Ʃp\times a(f)\times h)}}$

In this equation, ƩQ represents the sum of the counted osteoblasts for each sample, a(f) represents the frame area and Ʃp represents the total number of fields in X- and Y-axis on which osteoblasts were counted.

Kruskal-Wallis non-parametric test was used for data analysis and a *p* Value <0.05 was considered statistically significant. SPSS statistical software (version 15) was utilized for statistical analysis.

## Results

### Storage Media Comparison

Four groups of storage media were compared with each other sorted and layered by similar time interval and storage temperature, each group contained five specimens. Table 1 shows different storage media comparisons in different preservation temperatures and times.

1. Comparison of four storage media groups, which preserved bone grafts for 2 hours in cold temperature, showed that the highest count was noted in samples stored in autologous blood, followed by dry environment, Ringer’s lactate solution, and finally normal saline solution respectively (*p*< 0.05).

2. Comparison of four storage media groups which preserved bone grafts for 4 hours in cold temperature showed the highest count was noted in samples stored in normal saline solution, followed by autologous blood, dry environment and finally Ringer’s lactate solution respectively (*p*<0.05). 

3. Comparison of four storage media groups which preserved bone grafts for 12 hours in cold temperature showed that the highest count was noted in samples stored in dry environment, followed by Ringer’s lactate solution, normal saline solution, and autologous blood, respectively (*p*<0.05).

4. Comparison of four storage media groups, which preserved bone grafts for 2 hours in room temperature, showed the highest count was noted in samples stored in autologous blood, followed by ringer lactate or normal saline solutions and finally dry environment respectively (*p*< 0.05). No significant results were shown between samples stored in normal saline solution and Ringer’s lactate solution (*p*> 0.05). 

**Table 1:** Kruskal-Wallis comparison results for different storage media for bone graft preservation.

	Solution (I)	Solution (J)	2 hours		4 hours		12 hours	
			Mean Difference (I-J)	Sig.	Mean Difference (I-J)	Sig.	Mean Difference (I-J)	Sig.
Cold temperature	Saline	Ringer	-3955.92140*	.000	25196.30000*	.000	-24263.0960*	.000
		Blood	-59558.8714*	.000	11199.06000*	.000	6843.42000*	.000
		Dry	-43916.7214*	.000	17637.41000*	.000	-43860.2200*	.000
	Ringer	Saline	3955.92140*	.000	-25196.3000*	.000	24263.09600*	.000
		Blood	-55602.9500*	.000	-13997.2400*	.000	31106.51600*	.000
		Dry	-39960.8000*	.000	-7558.89000*	.000	-19597.1240*	.000
	Blood	Saline	59558.87140*	.000	-11199.0600*	.000	-6843.42000*	.000
		Ringer	55602.95000*	.000	13997.24000*	.000	-31106.5160*	.000
		Dry	15642.15000*	.000	6438.35000*	.000	-50703.6400*	.000
	Dry	Saline	43916.72140*	.000	-17637.4100*	.000	43860.22000*	.000
		Ringer	39960.80000*	.000	7558.89000*	.000	19597.12400*	.000
		Blood	-15642.1500*	.000	-6438.35000*	.000	50703.64000*	.000
Room temperature	Saline	Ringer	-.00020	1.000	.00060	1.000	-.05320	1.000
		Blood	-50540.8204*	.000	-70851.9100*	.000	-49492.7368*	.000
		Dry	3804.14960*	.000	-11804.9298*	.000	-59024.6604*	.000
	Ringer	Saline	.00020	1.000	-.00060	1.000	.05320	1.000
		Blood	-50540.8202*	.000	-70851.9106*	.000	-49492.6836*	.000
		Dry	3804.14980*	.000	-11804.9304*	.000	-59024.6072*	.000
	Blood	Saline	50540.82040*	.000	70851.91000*	.000	49492.73680*	.000
		Ringer	50540.82020*	.000	70851.91060*	.000	49492.68360*	.000
		Dry	54344.97000*	.000	59046.98020*	.000	-9531.92360*	.000
	Dry	Saline	-3804.14960*	.000	11804.92980*	.000	59024.66040*	.000
		Ringer	-3804.14980*	.000	11804.93040*	.000	59024.60720*	.000
		Blood	-54344.9700*	.000	-59046.9802*	.000	9531.92360*	.000
Incubator	Saline	Ringer	-11198.3490*	.000	15747.68000*	.000	33361.77040*	.000
		Blood	-16797.5278*	.000	1283.14220	.056	29862.28000*	.000
		Dry	-28891.7600*	.000	6415.72040*	.000	1866.40060*	.004
	Ringer	Saline	11198.34900*	.000	-15747.6800*	.000	-33361.7704*	.000
		Blood	-5599.17878*	.000	-14464.5378*	.000	-3499.49040*	.000
		Dry	-17693.4110*	.000	-9331.95960*	.000	-31495.3698*	.000
	Blood	Saline	16797.52778*	.000	-1283.14220	.056	-29862.2800*	.000
		Ringer	5599.17878*	.000	14464.53780*	.000	3499.49040*	.000
		Dry	-12094.2322*	.000	5132.57820*	.000	-27995.8794*	.000
	Dry	Saline	28891.76000*	.000	-6415.72040*	.000	-1866.40060*	.004
		Ringer	17693.41100*	.000	9331.95960*	.000	31495.36980*	.000
		Blood	12094.23222*	.000	-5132.57820*	.000	27995.87940*	.000

* Statistically meaningful difference(*p* <0.05)

5. Comparison of four storage media groups, which preserved bone grafts for 4 hours in room temperature, showed that the highest count was noted respectively in samples stored in autologous blood, followed by dry environment and finally ringer lactate or normal saline solutions (*p*< 0.05). No significant results were shown between samples stored in normal saline solution and Ringer’s lactate solution (*p*> 0.05). 

6. Comparison of four storage media groups, which preserved bone grafts for 12 hours in room temperature, showed the highest count was noted respectively in samples stored in dry environment, followed by autologous blood and finally ringer lactate or normal saline solutions (*p*< 0.05). No significant results were shown between samples stored in normal saline solution and Ringer’s lactate solution (*p*> 0.05). 

7. Comparison of four storage media groups which preserved bone grafts for 2 hours in incubator showed the highest count was noted respectively in samples stored in dry environment, followed by autologous blood, Ringer’s lactate solution, and finally normal saline solution (*p*< 0.05). 

8. Comparison of four storage media groups which preserved bone grafts for 4 hours in incubator showed the highest count was noted respectively in samples stored in autologous blood or normal saline solution, followed by dry environment and finally Ringer’s lactate solution (*p*< 0.05). No significant results were shown between samples stored in normal saline solution and autologous blood (*p*= 0.056). 

9. Comparison of four storage media groups, which preserved bone grafts for 12 hours in incubator showed the highest count was noted respectively in samples stored in normal saline solution, followed by dry environment, autologous blood, and finally Ringer’s lactate solution (*p*< 0.05). 

### Storage Temperature Comparison

Three groups of storage temperature were compared with each other, sorted and layered by similar time interval and storage medium; each group contained five specimens. Table 2 shows different storage temperatures comparisons in different storage media and times.

1. Comparison of three storage temperature groups, which preserved bone grafts for 2 hours in normal saline solution, showed the highest count was noted respectively in samples stored ‎in room temperature, followed by cold preservation and finally incubator (*p*< 0.05). 

2. Comparison of three storage temperature groups, which preserved bone grafts for 4 hours in normal saline solution, showed the highest count was noted respectively in samples stored ‎in cold preservation, followed by room temperature and finally incubator (*p*< 0.05). 

3. Comparison of three storage temperature groups, which preserved bone grafts for 12 hours in normal saline solution, showed the highest count was noted respectively in samples stored ‎in incubator, followed by cold preservation and finally room temperature (*p*< 0.05). 

4. Comparison of three storage temperature groups, which preserved bone grafts for 2 hours in Ringer’s lactate solution, showed the highest count was noted respectively in samples stored ‎in room temperature, followed by incubator and finally cold preservation (*p*< 0.05).

5. Comparison of three storage temperature groups, which preserved bone grafts for 4 hours in Ringer’s lactate solution, showed the highest count was noted respectively in samples stored ‎in room temperature, followed by cold preservation and finally incubator (*p*< 0.05). 

**Table 2:** Kruskal-Wallis comparison results for different temperatures for bone graft preservation.

	Temperature (I)	Temperature (J)	2 hours		4 hours		12 hours	
			Mean Difference(I-J)	Sig.	Mean Difference (I-J)	Sig.	Mean Difference (I-J)	Sig.
Normal saline solution	2-8^°c^	18-24^°c^	-31158.59100*	.000	10965.05000*	.000	2566.29120*	.000
		37^°c^	3276.34860*	.000	12248.20000*	.000	-20530.32000*	.000
	18-24^°c^	2-8^°c^	31158.59100*	.000	-10965.05000*	.000	-2566.29120*	.000
		37^°c^	34434.93960*	.000	1283.15000*	.048	-23096.61120*	.000
	37^°c^	2-8^°c^	-3276.34860*	.000	-12248.20000*	.000	20530.32000*	.000
		18-24^°c^	-34434.93960*	.000	-1283.15000*	.048	23096.61120*	.000
Ringer’s lactate solution	2-8^°c^	18-24^°c^	-27202.66980*	.000	-14231.24940*	.000	26829.33400*	.000
		37^°c^	-3966.07900*	.000	2799.58000*	.000	37094.54640*	.000
	18-24^°c^	2-8^°c^	27202.66980*	.000	14231.24940*	.000	-26829.33400*	.000
		37^°c^	23236.59080*	.000	17030.82940*	.000	10265.21240*	.000
	37^°c^	2-8^°c^	3966.07900*	.000	-2799.58000*	.000	-37094.54640*	.000
		18-24^°c^	-23236.59080*	.000	-17030.82940*	.000	-10265.21240*	.000
Autologous blood	2-8^°c^	18-24^°c^	-22140.54000*	.000	-71085.92000*	.000	-53769.86560*	.000
		37^°c^	46037.69222*	.000	2332.28220*	.000	2488.54000*	.000
	18-24^°c^	2-8^°c^	22140.54000*	.000	71085.92000*	.000	53769.86560*	.000
		37^°c^	68178.23222*	.000	73418.20220*	.000	56258.40560*	.000
	37^°c^	2-8^°c^	-46037.69222*	.000	-2332.28220*	.000	-2488.54000*	.000
		18-24^°c^	-68178.23222*	.000	-73418.20220*	.000	-56258.40560*	.000
Dry environment	2-8^°c^	18-24^°c^	16562.28000*	.000	-18477.28980*	.000	-12598.14920*	.000
		37^°c^	18301.31000*	.000	1026.51040*	.035	25196.30060*	.000
	18-24^°c^	2-8^°c^	-16562.28000*	.000	18477.28980*	.000	12598.14920*	.000
		37^°c^	1739.03000*	.004	19503.80020*	.000	37794.44980*	.000
	37^°c^	2-8^°c^	-18301.31000*	.000	-1026.51040*	.035	-25196.30060*	.000
		18-24^°c^	-1739.03000*	.004	-19503.80020*	.000	-37794.44980*	.000

* Statistically meaning full Difference (*p* <0.05)

6. Comparison of three storage temperature groups, which preserved bone grafts for 12 hours in Ringer’s lactate solution, showed the highest count was noted respectively in samples stored ‎in cold preservation, followed by room temperature and finally incubator (*p*< 0.05). 

7. Comparison of three storage temperature groups, which preserved bone grafts for 2 hours in autologous blood, showed the highest count was noted respectively in samples stored ‎in room temperature, followed by cold preservation and finally incubator (*p*< 0.05). 

8. Comparison of three storage temperature groups, which preserved bone grafts for 4 hours in autologous blood, showed the highest count was noted respectively in samples stored ‎in room temperature, followed by cold preservation and finally incubator (*p*< 0.05). 

9. Comparison of three storage temperature groups, which preserved bone grafts for 12 hours in autologous blood, showed the highest count was noted respectively in samples stored ‎in room temperature, followed by cold preservation and finally incubator (*p*< 0.05). 

10. Comparison of three storage temperature groups, which preserved bone grafts for 2 hours in dry environment, showed the highest count was noted respectively in samples stored ‎in cold preservation, followed by room temperature and finally incubator (*p*< 0.05). 

11. Comparison of three storage temperature groups, which preserved bone grafts for 4 hours in dry environment, showed the highest count was noted respectively in samples stored ‎in room temperature, followed by cold preservation and finally incubator (*p*< 0.05). 

12. Comparison of three storage temperature groups, which preserved bone grafts for 12 hours in dry environment, showed the highest count was noted respectively in samples stored ‎in room temperature, followed by cold preservation and finally incubator (*p*< 0.05). 

### Storage Time Comparison

Three groups of storage time were compared with each other sorted and layered by similar storage temperature and medium, each group contained five specimens. Table 3 shows different storage times comparison in different preservation media and temperatures.

**Table 3:** Kruskal-Wallis comparison results for different storage times for bone graft preservation.

	Time (I)	Time (J)	Cold preservation		Room temperature		Incubator	
			Mean Difference (I-J)	Sig.	Mean Difference (I-J)	Sig.	Mean Difference (I-J)	Sig.
Normal saline solution	2.00	4.00	-30318.7114*	.000	11804.92960*	.000	-21346.8600*	.000
		12.00	-11654.7914*	.000	22070.09080*	.000	-35461.4600*	.000
	4.00	2.00	30318.71140*	.000	-11804.9296*	.000	21346.86000*	.000
		12.00	18663.92000*	.000	10265.16120*	.000	-14114.6000*	.000
	12.00	2.00	11654.79140*	.000	-22070.0908*	.000	35461.46000*	.000
		4.00	-18663.9200*	.000	-10265.1612*	.000	14114.60000*	.000
Ringer’s lactate solution	2.00	4.00	-1166.49000*	.000	11804.93040*	.000	5599.16900*	.000
		12.00	-31961.9660*	.000	22070.03780*	.000	9098.65940*	.000
	4.00	2.00	1166.49000*	.000	-11804.9304*	.000	-5599.16900*	.000
		12.00	-30795.4760*	.000	10265.10740*	.000	3499.49040*	.000
	12.00	2.00	31961.96600*	.000	-22070.0378*	.000	-9098.65940*	.000
		4.00	30795.47600*	.000	-10265.1074*	.000	-3499.49040*	.000
Autologous blood	2.00	4.00	40439.22000*	.000	-8506.16000*	.000	-3266.19002*	.000
		12.00	54747.50000*	.000	23118.17440*	.000	11198.34778*	.000
	4.00	2.00	-40439.2200*	.000	8506.16000*	.000	3266.19002*	.000
		12.00	14308.28000*	.000	31624.33440*	.000	14464.53780*	.000
	12.00	2.00	-54747.5000*	.000	-23118.1744*	.000	-11198.3478*	.000
		4.00	-14308.2800*	.000	-31624.3344*	.000	-14464.5378*	.000
Dry environment	2.00	4.00	31235.42000*	.000	-3804.14980*	.000	13960.62040*	.000
		12.00	-11598.2900*	.000	-40758.7192*	.000	-4703.29940*	.000
	4.00	2.00	-31235.4200*	.000	3804.14980*	.000	-13960.6204*	.000
		12.00	-42833.7100*	.000	-36954.5694*	.000	-18663.9198*	.000
	12.00	2.00	11598.29000*	.000	40758.71920*	.000	4703.29940*	.000
		4.00	42833.71000*	.000	36954.56940*	.000	18663.91980*	.000

Statistically meaning full Difference (*p* <0.05)

1. Comparison of three time-period groups, which stored bone grafts in normal saline solution in cold preservation, showed the highest count was noted respectively in samples ‎stored for 4 hours, followed by 12 hours and finally 2 hours (*p*< 0.05). 

2. Comparison of three time-period groups, which stored bone grafts in Ringer’s lactate solution in cold preservation, showed the highest count was noted respectively in samples ‎stored for 12 hours, followed by 4 hours and finally 2 hours (*p*< 0.05). 

3. Comparison of three time-period groups, which stored bone grafts in autologous blood in cold preservation, showed the highest count was noted respectively in samples ‎stored for 2 hours, followed by 4 hours and finally 12 hours (*p*< 0.05). 

4. Comparison of three time-period groups, which stored bone grafts in dry environment in cold preservation, showed the highest count was noted respectively in samples ‎stored for 12 hours, followed by 2 hours and finally 4 hours (*p*< 0.05). 

5. Comparison of three time-period groups, which stored bone grafts in normal saline solution in room temperature, showed the highest count was noted respectively in samples ‎stored for 2 hours, followed by 4 hours and finally 12 hours (*p*< 0.05). 

6. Comparison of three time-period groups, which stored bone grafts in Ringer’s lactate solution in room ‎ temperature, showed the highest count was noted respectively in samples ‎stored for 2 hours, followed by 4 hours and finally 12 hours (*p*< 0.05). 

7. Comparison of three time-period groups, which stored bone grafts in autologous blood in room ‎ temperature, showed the highest count was noted respectively in samples ‎stored for 4 hours, followed by 2 hours and finally 12 hours (*p*< 0.05). 

8. Comparison of three time-period groups, which stored bone grafts in dry environment in room ‎ temperature, showed the highest count was noted respectively in samples ‎stored for 12 hours, followed by 4 hours and finally 2 hours (*p*< 0.05).

9. Comparison of three time-period groups, which stored bone grafts in normal saline solution in incubator, showed the highest count was noted respectively in samples ‎stored for 12 hours, followed by 4 hours and finally 2 hours (*p*< 0.05). Comparison of three time-period groups, which stored bone grafts in Ringer’s lactate solution in incubator, showed the highest count was noted respectively in samples ‎stored for 2 hours, followed by 4 hours and finally 12 hours (*p*< 0.05). 

10. Comparison of three time-period groups, which stored bone grafts in autologous blood in incubator, showed the highest count was noted respectively in samples ‎stored for 4 hours, followed by 2 hours and finally 12 hours (*p*< 0.05). 

11. Comparison of three time-period groups, which stored bone grafts in dry environment in incubator, showed the highest count was noted respectively I samples ‎stored for 12 hours, followed by 2 hours and finally 4 hours (*p*< 0.05). 

## Discussion

The preservation circumstances studied in this article included different times, different storage media, and different storage temperatures. The time interval the grafts are preserved extracorporeally is a result of surgery and patient’s condition, but the storage medium and temperature is a choice the surgeon can decide. Based on the time expected for the surgery, one can determine the best storage condition for the highest quality of grafts.

In comparing the different storage media, in all specimens stored in room temperature, Ringer’s lactate solution and normal saline solution showed no difference in osteoblast preservation. In all grafts stored for 2 hours, autologous blood was superior to Ringer’s lactate solution and normal saline solution. In addition, in all bone grafts preserved for 12 hours, those stored in dry environment showed higher count of osteoblasts than that in Ringer’s lactate solution and blood.

In a total comparison, blood was a better medium than Ringer’s lactate solution, except grafts stored for 12 hours in cold preservation. In addition, dry environment preserved more osteoblasts in all situations, except for preservation of grafts for 2 hours in room temperature. 

In the bone grafts stored for 2 hours in room temperature, the autologous blood resulted in the highest count of osteoblast, followed by ringer lactate or normal saline solution with dry environment causing the least count of osteoblasts. This is similar to the study Rocha et al. [ 8 ] performed, considering that they only stored the grafts for 30 minutes in room temperature, and found dry environment causing more empty lacunas. They found no difference between the grafts implanted immediately after harvesting and those stored in room temperature in either normal saline solution or PPP.

In comparing different temperatures, in all grafts stored for 4 hours, the ones kept in incubator showed the lowest osteoblast count; those stored in dry environment and blood all showed the same result. The specimens stored in autologous blood, room temperature was the best in preserving osteoblasts.

In a total comparison, incubator temperature caused the least count of osteoblasts except for the grafts stored for 2 hours in Ringer’s lactate solution and for 12 hours in normal saline solution. Moreover, in all specimens except those kept for 2 hours in dry environment, 4 hours in normal saline solution and 12 hours in either normal saline or Ringer’s lactate solution, room temperature was the best storage temperature for osteoblast preservation.

Antonenas et al. [ 9 ] showed more viable cells in grafts stored in normal saline solution in cold preservation than those kept in room temperature for 24, 48 or 72 hours; in this study, the specimens stored in normal saline for 4 or 12 hours, showed higher count of osteoblast in cold preservation than room temperature. 

In comparing different time-periods the grafts were kept, in those preserved in room temperature, the grafts that were stored for 12 hours showed the least count of osteoblasts, except for the specimens stored in dry environment. In all bone grafts stored in autologous blood, those preserved for 12 hours showed less count of osteoblasts than those kept for 2 or 4 hours. However, the graft particles, which were stored for 12 hours in dry environment, showed higher count of osteoblast in comparison with those, preserved for 2 or 4 hours. 

William et al. [ 10 ] preserved osteochondral grafts in refrigerator for at 4^°c^ for 14, 21 and 28 days. Their study showed> 95% cell viability at 14 days, 75-98% at 21 days and 65-90% at 28 days of preservation. They stored osteochondral femoral condyles in a solution containing 10% fetal calf serum, glutamate, non-essential amino acids and anti-microbial agents like penicillin, streptomycin, and fungizone. Similarly, in our study the grafts in any medium, either infusion solutions or blood showed the least count of osteoblasts in 12 hours of storage than those stored for 2 or 4 hours. 

In future studies, we recommend a further in vivo analysis to correlate these in vitro results with graft survival chance. In addition, other criteria such as bone spicule density can correlate with less bone graft resorption, which should be evaluated. One must consider that bone graft surgery success depends on many factors other than extracorporeal storage method, including surgeons’ qualification, soft tissue handling, recipient site quality, patient’s general health condition, and so on; all these factors may influence the results of in vivo studies.

## Conclusion

In a total comparison, blood is a better medium than Ringer’s lactate solution, except for the grafts stored for 12 hours in cold preservation. The specimens stored in autologous blood, room temperature were the best in preserving the osteoblasts. In all bone grafts stored in autologous blood, those preserved for 12 hours showed less number of osteoblasts compared to those kept for 2 or 4 hours. Based on the results from this study, in order to preserve the highest count of osteoblasts in bone graft extracorporeal storage, room temperature and autologous blood is recommended. Room temperature is the most favorable and recommended temperature and the favorable storage media are saline solution, Ringer lactate solution, and autogenous blood. Moreover, the best time for bone storage out of body is shorter than 4 hours and longer preservation time would result in noticeable loss of osteoblasts, therefore it should be avoided.
